# Anti-tumoral efficacy of therapeutic human anti-KIR antibody (Lirilumab/BMS-986015/IPH2102) in a preclinical xenograft tumor model

**DOI:** 10.1186/2051-1426-1-S1-P40

**Published:** 2013-11-07

**Authors:** Caroline Sola, Fabien Chanuc, Ariane Thielens, Nicolas Fuseri, Yannis Morel, Mathieu Bléry, Pascale André, Eric Vivier, Robert Graziano, Francois Romagne, Cécile Bonnafous

**Affiliations:** 1 Innate Pharma, Marseille, France; 2 Centre d'Immunologie de Marseille-Luminy, Université d'Aix-Marseille, Marseille, France; 3 Bristol-Myers Squibb, Princeton, NJ, USA

## 

Natural Killer cells (NK cells) are lymphocytes able to recognize and kill tumors for which the expression of Major Histocompatibility Complex (MHC) class I molecules is altered. This “missing self” recognition is mediated in humans by the lack of engagement of MHC class I i.e. Human Leucocyte Antigens (HLA) molecules with NK cell inhibitory receptors that include Killer Immunoglobulin-like Receptors (KIR). Some tumors escape NK cell immune surveillance by increasing the expression of HLA molecules on their surface. Consequently, blocking interactions between KIR and HLA molecules constitutes an interesting therapeutic strategy. The anti-KIR2DL1/2/3-specific monoclonal antibody, lirilumab/BMS-986015/IPH2102, is a human IgG4 that is being developed for treating both hematologic malignancies and solid tumors. In rodents, the MHC class I inhibitory system regulating NK cell activation is based on lectin-like family Ly49 but the KIR molecules are not expressed. The objective of this study was to develop a preclinical model to assess the efficacy of the drug candidate used in clinical trials, lirilumab. Mice expressing the human NK inhibitory KIR2DL3, on the surface of NK cells were generated on a RAG-1deficient background (KIRtgRAG mice). The human B cell lymphoma cell line, 721.221, transduced with HLA-Cw3 molecule, a ligand of KIR2DL3, was intra-venously engrafted in these mice. The expression of HLA-C by tumor cells was sufficient to allow them to escape control of NK cells, leading to mice death in around 30 days. Lirilumab treatment increased mice survival in a dose dependent manner when injected at the same time as the tumor challenge. This protective effect was NK cell-mediated and directly correlated with the duration of KIR saturation. Interestingly, lirilumab treatment also improved survival in therapeutic conditions i.e. when the antibody was injected 5 days after the tumor, also in a NK cell-dependent manner. In conclusion, this study showed efficacy of lirilumab as single agent in a HLA-Cw3-expressing tumor model and this xenogenic pre-clinical model will be an excellent tool to investigate the therapeutic benefits of combination treatments.

